# Flow-cytometric assessment of cellular poly(ADP-ribosyl)ation capacity in peripheral blood lymphocytes

**DOI:** 10.1186/1742-4933-3-8

**Published:** 2006-07-19

**Authors:** Andrea Kunzmann, Dan Liu, Kathryn Annett, Muriel Malaisé, Bastian Thaa, Paul Hyland, Yvonne Barnett, Alexander Bürkle

**Affiliations:** 1 Molecular Toxicology Group, Department of Biology, Box X911, University of Konstanz, D-78457 Konstanz, Germany; 2 Cancer and Ageing Research Group, School of Biomedical Sciences, University of Ulster, Coleraine, BT52 1SA, Northern Ireland, UK; 3 School of Biomedical and Natural Sciences, College of Science and Technology, Nottingham Trent University, Clifton Campus, NG11 8NS, Nottingham, UK

## Abstract

**Background:**

Poly(ADP-ribosyl)ation is a posttranslational modification of nuclear proteins catalysed by poly(ADP-ribose) polymerases (PARPs), using NAD^+ ^as a substrate. Activation of PARP-1 is in immediate response to DNA damage generated by endogenous and exogenous damaging agents. It has been implicated in several crucial cellular processes including DNA repair and maintenance of genomic stability, which are both intimately linked with the ageing process. The measurement of cellular poly(ADP-ribosyl)ation capacity, defined as the amount of poly(ADP-ribose) produced under maximal stimulation, is therefore relevant for research on ageing, as well as for a variety of other scientific questions.

**Results:**

This paper reports a new, robust protocol for the measurement of cellular poly(ADP-ribosyl)ation capacity in PBMC or Jurkat T-cells using flow cytometry, based on a previously established immuno-dot-blot assay. In order to validate the new assay, we determined the dose-response curve of 3-aminobenzamide, a well-known competitive PARP inhibitor, and we derived an IC_50 _that is very close to the published value. When testing a set of PBMC samples taken from fifteen healthy young human donors, we could confirm the presence of a substantial interindividual variation, as previously observed using a radiometric assay.

**Conclusion:**

The methodology described in this paper should be generally useful for the determination of cellular poly(ADP-ribosyl)ation capacity in a wide variety of settings, especially for the comparison of large sets of samples, such as population studies. In contrast to previously published radiometric or immuno-dot-blot assays, the new FACS-based method allows (i) selective analysis of mononuclear cells by gating and (ii) detection of a possible heterogeneity in poly(ADP-ribosyl)ation capacity between cells of the same type.

## Background

Ageing is a multifactorial degenerative process that affects all tissues including the immune system [1]. There is evidence for a loss of genomic stability in cells during normal ageing (for review: [2,3]). This genomic instability may well be mediated by limitations in DNA repair pathways. This view is supported by recent reports highlighting the pivotal role of DNA repair (*e.g*. nucleotide excision repair) in determining the rate of ageing [4], while on the other hand proteins that are found deficient in syndromes of accelerated ageing, such as the Werner protein (WRN), have been shown to possess functions in DNA repair and maintenance of genomic stability (for review: [2]).

The bioavailability and intracellular distribution of zinc ions may well have an impact on processes related with DNA repair and maintenance of genomic stability, and thus on the ageing process [5]. This is apparent from the fact that several DNA repair-related proteins are zinc-finger proteins [6]. One prominent example of a zinc finger protein involved in DNA repair and genomic stability is poly(ADP-ribose) polymerase-1 (PARP-1, EC 2.4.2.30). PARP-1 catalyses one of the immediate cellular responses to genotoxic stress, *i.e*. the synthesis of poly(ADP-ribose) [3,7-10]. On the one hand, this enzyme is a very promising target for cancer chemotherapy [11], and especially so for BRCA2-deficient tumour cells [12]. On the other hand, an involvement of poly(ADP-ribose) metabolism in the ageing process has long been suggested, as we could show that the cellular capacity to produce poly(ADP-ribose) in peripheral blood mononuclear cells (PBMC) correlates positively with species-specific life span in mammals [13]. Furthermore, we were able to establish an association between high cellular poly(ADP-ribosyl)ation capacity in lymphoblastoid cells with human longevity [14].

Environmental toxins that can interfere with the structural integrity of zinc fingers, such as arsenicals, have recently been shown to suppress DNA damage-induced poly(ADP-ribose) formation in living cells in culture, even at remarkably low concentrations that prevail in drinking water from some geographical regions of the world [15]. Other conditions that might lead to similar effects are oxidative protein damage or diminished bioavailability of zinc, resulting from either nutritional zinc deficiency or changes in zinc transport or intracellular storage.

One of the tasks of the ZINCAGE project [5] supported by the EU Commission is, therefore, to assess poly(ADP-ribosyl)ation capacity in human PBMC as a function of age and nutritional zinc status of the donor. The data obtained will be correlated with series of other genetic, biochemical and psychological parameters to be assessed within ZINCAGE. Based on the importance and multi-functional nature of poly(ADP-ribosyl)ation, it is obvious that reliable and convenient methods to assess cellular poly(ADP-ribosyl)ation capacities are needed.

Previously, various methods have been developed to assess the cellular poly(ADP-ribosyl)ation capacity. This was primarily achieved by incubating permeabilised cells with a double-stranded activator DNA oligonucleotide and then subsequently measuring the incorporation of radiolabelled NAD^+ ^into acid-insoluble material [13,14,16]. There were, however, major drawbacks with that methodology including the requirement of using radioactivity, of relatively large cell numbers, and the notorious inefficiency of the washing steps to remove any unincorporated radioactively labelled NAD^+ ^from trichloroacetic acid (TCA) precipitates. Therefore, we subsequently developed a non-radioactive immuno-dot-blot assay to quantify cellular poly(ADP-ribosyl)ation capacity [17]. That method allowed permeabilised cells that had been incubated with an activator oligonucleotide and non-labelled NAD^+ ^to be directly dot-blotted onto a nylon membrane, and then TCA-precipitated *in situ*. Immunodetection of the resultant poly(ADP-ribose) was achieved using a monoclonal antibody conjugated with a peroxidase-based quantitative chemiluminescence detection system. That method, however, did not allow separation of specific cell types or detection of heterogeneity between cells of the same type.

In the present paper we describe a new methodology using a flow cytometry-based technique.

## Results and discussion

In this study, a modification to an existing dot-blot assay for the quantification of cellular poly(ADP-ribosyl)ation capacity [17] was developed based on flow cytometry.

**Figure 1:**
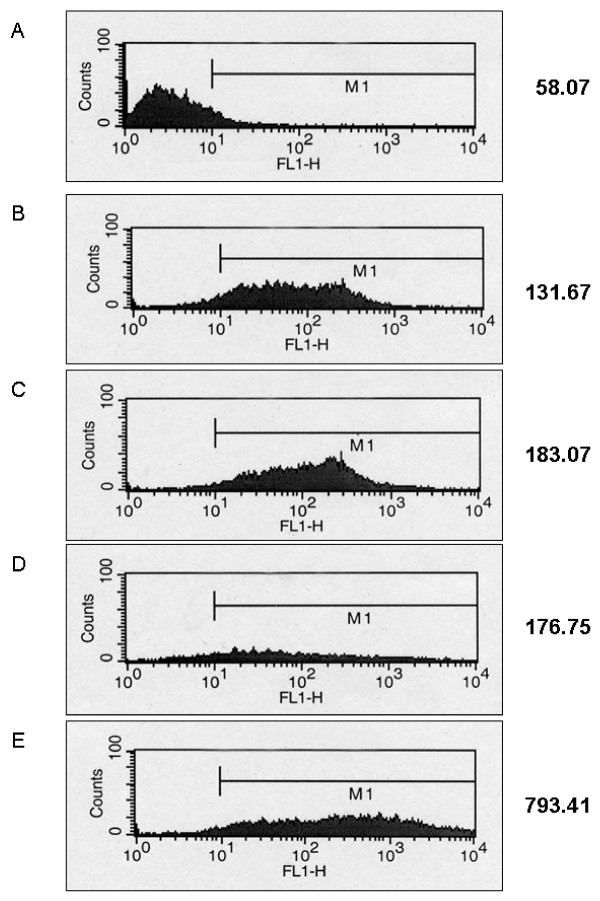
**Frequency distributions (histograms) of MFI representing poly(ADP-ribosyl)ation levels in permeabilised Jurkat cells**. (A) Untreated cells, no antibodies (Ab) added (negative control); (B) no NAD^+ ^and no oligo in the reaction buffer; primary Ab, secondary Ab; (C) no NAD^+^, but oligo in the reaction buffer; primary Ab, secondary Ab; (D) NAD^+^, but no oligo in the reaction buffer; primary Ab, secondary Ab; (E) NAD^+ ^and oligo in the reaction buffer; primary Ab, secondary Ab. Note that a shift to the right (FL-1; x-axis) indicates increased levels of poly(ADP-ribosyl)ation. Numerical values next to the histograms represent the mean fluorescence intensity (MFI). M1, intensity range used to determine mean of FL1-H.

In order to assess levels of cellular poly(ADP-ribosyl)ation by such a method, background levels of mean fluorescence intensity (MFI) had to be established. Initial experiments were carried out using Protocol A to determine MFI levels in untreated Jurkat T cells, *i.e*. cells with no antibody labelling, serving as a negative control and regarded as a measure for background fluorescence. In addition, antibody staining was performed on permeabilised cells incubated with or without NAD^+ ^in the presence or absence of activator oligonucleotide. Figure 1 shows the typical frequency distributions (histograms) obtained with Protocol A. Figure 1(A) represents the negative control (MFI = 58.07), and can be regarded as background fluorescence level as these cells were not antibody-labelled. Panels (B) – (D) show the histograms of Jurkat T cell samples stained with primary and secondary antibodies after treatment of the cells as detailed in the protocol, except for the lack of both NAD^+ ^and the activator oligonucleotide (B), or the lack of NAD^+ ^only (C), or the lack of oligonucleotide only (D). In panels B – D a slight increase in MFI is apparent, ranging between approximately 130 and 180 units, reflecting more or less the background staining of the primary and secondary antibody, as no significant amounts of poly(ADP-ribose) were produced under these conditions. Permeabilised cells, however, that had been incubated with NAD^+ ^plus activator oligonucleotide and stained with antibodies (Panel E) yielded a severalfold higher fluorescence intensity (MFI = 793.41), as expected, because PARP-1 was fully stimulated under these conditions. This result clearly indicates the usefulness of flow cytometry as tool for assessment of poly(ADP-ribosyl)ation capacity.

In the course of our experiments we developed a modified assay protocol (Protocol B), for measuring PBMC. The main differences between Protocol A and Protocol B are (i) fixation/permeabilisation ("prefixation ") with 100% ethanol before the reaction is started, and (ii) a second fixation step using formaldehyde to be performed after the PARP-1 reaction has taken place. The prefixation is done to "stabilise" the cells for subsequent steps of the experimental procedure. It should be noted that DNA strand break-driven poly(ADP-ribosyl)ation occurs mainly as an automodification of PARP-1 [18]. Polymer formation leads to a strongly negatively charged protein modification. This can lead to a repulsion effect as DNA is also negatively charged, and therefore automodified PARP-1 might be lost from chromatin. Therefore a second fixation step was introduced using formaldehyde. This modified assay turned out to be more stable and reproducible and is therefore the recommended assay format.

**Figure 2:**
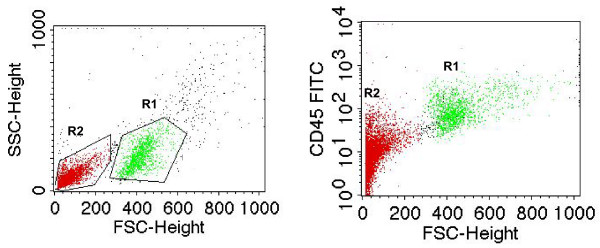
**Separation of leukocytes by CD 45 staining and gating**. (A) Gating of cells and classification as populations R1 and R2. (B) PBMC were stained with CD 45 antibody. The R1 population is CD 45-positive.

Flow cytometry provides the option of selecting certain cell populations by gating. For example, we are interested in measuring PARP activity specifically in PBMC and therefore wish to exclude other cell types (such as platelets) contaminating the preparation. PBMC can be stained with a CD 45 antibody and selected by gating as is depicted in Figure 2. This cell population was selectively analysed in all subsequent experiments.

**Figure 3:**
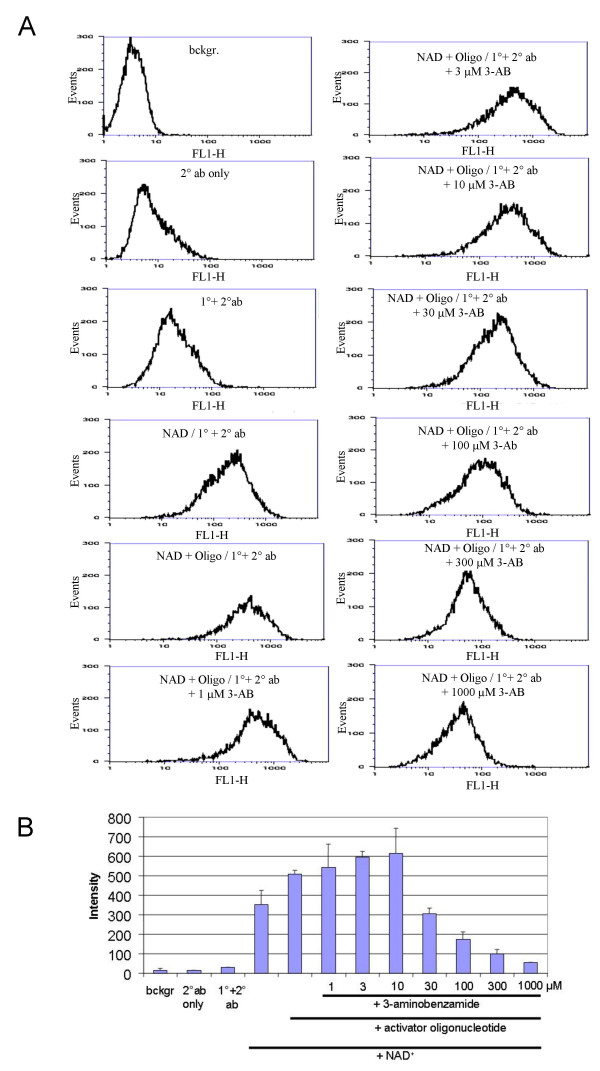
**Stimulation of PARP activity in permeabilised human PBMC by addition of NAD and activator oligonucleotide, and inhibitory effect of 3-aminobenzamide**. A. Histograms of poly(ADP-ribosyl)ation levels determined using Protocol B. Permeabilised cells were incubated with NAD^+ ^and activator oligonucleotide in the presence of the PARP inhibitor 3-aminobenzamide as indicated. A representative set of data is shown. bckgr, background; ab, antibody; 3-AB, 3-aminobenzamide. Note that a shift to the right (FL-1; x-axis) indicates increased levels of poly(ADP-ribosyl)ation and that addition of PARP inhibitor 3-aminobenzamide reverses this shift. B. Quantitative evaluation of MFI. Shown are average values and SD of three parallel determinations, respectively, comprising the data set shown in A

As is depicted in Figure 3, MFI values of human PBMC incubated in the absence of NAD^+ ^were very low, whereas addition of NAD^+ ^and, even more so, further addition of activator oligonucleotide raised MFI by over tenfold. To illustrate the usefulness of the flow cytometry-based assay according to Protocol B, the well-known PARP inhibitor 3-aminobenzamide was added to the reaction mixture at varying concentrations, i.e. 1, 3, 10, 30, 100, 300, and 1,000 μM (Fig. 3). As expected, a significant and concentration-dependent inhibition of poly(ADP-ribose) formation was observed at concentrations between 30 and 1,000 μM, down to background levels at the highest concentration tested, thus demonstrating the specificity of the immunostaining for poly(ADP-ribose). No significant change was seen at 3-aminobenzamide concentration up to 10 μM. The potency of the inhibitor observed in these experiments corresponds very well with its known IC_50 _*in vitro *(approximately 40 μM; [19]).

In previous work using a radiometric PARP activity assay [13], we had observed significant interindividual heterogeneity between samples taken from young healthy donors. This was the case for PBMC taken from any of the mammalian species tested. At least to some extent that heterogeneity may have been due to technical problems related with inefficient removal of non-incorporated radioactive NAD^+^. We therefore tested a set of PBMC samples taken from fifteen healthy human donors (24–29 years of age) using the new assay format (Table 1a). The average activity level (i.e., [MFI with NAD^+ ^and oligonucleotide] – [MFI without NAD^+^] was 665 with a standard deviation of 419. These data reveal a substantial (several-fold) interindividual variation, with no obvious gender difference. We also observed such variability between donors tested in parallel (Table 1a, donors marked with asterisks). This, however, was not due to instability of the assay, as measurements of PBMC from a single donor performed either in parallel or in consecutive experiments on different days displayed only marginal variability (Table 1b). Thus the variability seen in Table 1a seems to be biological rather than technical. Nevertheless, for large studies it is advisable to use internal standards like aliquots of cryopreserved PBMC from a single donor. It will be interesting to decipher the reasons for the pronounced interindividual variation as well as the possible biological consequences for the individual. One candidate, on which we are focussing in the ZINCAGE project, is the cellular zinc status.

**Table 1a:** Heterogeneity of poly(ADP-ribosyl)ation capacity of PBMC from healthy young human donors.

**Donor #**	**Age**	**Gender**	**MFI**
1*	29	m	601
2	29	m	410
3	28	m	914
4	28	m	546
5	27	m	283
6	26	m	1467
7	26	m	292
8*	29	f	1664
9*	29	f	462
10	29	f	433
11	28	f	793
12	27	f	838
13	27	f	553
14*	27	f	512
15	24	f	211
			
Average			665
SD			419

Cellular poly(ADP-ribosyl)ation capacity in PBMC of 15 young and healthy donors was measured using Protocol B. Note the high level of variability between donors. High variability was also noticed in a subset of samples processed in parallel in the same experiment (asterisks), with an average of 809 and SD of 572. MFI, mean fluorescence intensity; SD, standard deviation.

**Table 1b:** Robustness of poly(ADP-ribosyl)ation capacity measurements of PBMC from one donor done either in parallel or consecutive assays.

**parallel measurements**	**MFI**
	220
	190
	165
	269
	210
	197
**Average**	**208**
**SD**	**35**
	

**consecutive measurements**	**MFI**

	193
	201
	323
**Average**	**239**
**SD**	**73**

Cellular poly(ADP-ribosyl)ation capacity was measured using Protocol B. Top: Six independent reactions were started with PBMC from one donor within a single experiment. Bottom: The poly(ADP-ribosyl)ation capacity of one donor was measured in PBMC from blood samples obtained on different days. Note the relatively small variability of the measurements in contrast to samples from different donors. MFI, mean fluorescence intensity; SD, standard deviation.

## Conclusion

The methodology described in this paper should be generally useful for the determination of cellular poly(ADP-ribosyl)ation capacity in a wide variety of settings, especially for the comparison of large sets of samples, such as population studies. In contrast to previously published radiometric or immuno-dot-blot assays, the new FACS-based method allows (i) selective analysis of mononuclear cells by gating and (ii) detection of a possible heterogeneity in poly(ADP-ribosyl)ation capacity between cells of the same type.

## Methods

### Materials

Phosphate Buffered Saline (Dulbecco A; PBS) was from Oxoid, Basingstoke, UK. Foetal calf serum (FCS) was from Invitrogen, Paisley, UK. Tris hydrochloride (Tris-HCl), ethylenediaminetetraacetic acid (EDTA), magnesium chloride (MgCl_2_), 2-mercaptoethanol, digitonin, β-nicotinamide adenine dinucleotide (NAD^+^; grade V), 3-aminobenzamide (3-AB), sodium azide were from Sigma, Poole, UK. The PARP activator deoxyoligonucleotide (GGAATTCC) [16] was dissolved in 15 mM NaCl at 385 μg/ml. Percoll was from Amersham Bioscience, Uppsala, Sweden. Mouse monoclonal antibody recognising poly(ADP-ribose) was purified as described previously, from culture supernatant of 10 H hybridoma cells [20] (kind gift of M. Miwa and T. Sugimura, Tokyo, Japan) using a protein-A column chromatography kit [17]. Alexa Fluor^®^488 goat anti-mouse secondary antibody was from Molecular Probes, Paisley, UK. CD 45 antibody was from BD Bioscience, Erembodegem, Belgium.

### Cells

Jurkat T cells, a leukaemic T cell line, were maintained as a suspension culture in RPMI 1640 medium (Sigma) supplemented with 100 U/ml penicillin, 100 μg/ml streptomycin, 2 mM L-glutamine and 10 % foetal calf serum (Sigma). Cultures were incubated at 37°C and 5 % CO_2_.

Freshly obtained human PBMC from healthy donors were isolated by Percoll gradient centrifugation [13].

### FACS-based PARP-activity assay – Protocol A

To perform PARP activity assays, a modification of the dot blot method according to Pfeiffer et al. [17] was used.

Jurkat T cells were counted, washed in PBS, pelleted and resuspended in ice-cold hypotonic permeabilisation buffer (10 mM Tris-HCl pH 7.8, 1 mM EDTA, 4 mM MgCl_2_, 30 mM 2-mercaptoethanol) supplemented with 0.015% (w/v) digitonin (Sigma), and left on ice for 1 minute. A further 4 ml of digitonin-free, ice-cold permeabilisation buffer was then added and the cells were pelleted at 326 *g*, 4°C, for 10 minutes and resuspended again in ice-cold permeabilisation buffer at a density of 2 × 10^5 ^cells/53 μl in a V-bottomed 96-well plate.

In order to investigate cellular poly(ADP-ribosyl)ation capacity, the permeabilised Jurkat T cells were incubated with activator oligonucleotide and non-labelled NAD^+^, which acts as substrate, to allow formation of poly(ADP-ribose).

Thirty-four μl of reaction buffer (100 mM Tris-HCl pH 7.8, 1 mM NAD^+^, 120 mM MgCl_2_) and 13 μl of double-stranded activator oligonucleotide dissolved in 15 mM NaCl at 385 μg/ml was added to samples of 2 × 10^5 ^cells on ice, yielding a total volume of 100 μl per reaction. The mixture was incubated at 37°C for 4 minutes, and then cells were pelleted at 652 *g*, 4°C, for 3 minutes and resuspended in 60 μl PBS. The reaction was stopped by adding 140 μl of ice-cold 70% ethanol and incubation for 15 minutes at -20°C. Then cells were pelleted at 652 *g*, 4°C, for 5 minutes and then washed in 240 μl of FACS buffer (1 × PBS, 0.5% FCS, 2 mM NaN_3_), with shaking at 4°C, and again centrifuged at 652 *g*, 4°C for 2 minutes.

The cell pellet was resuspended in 240 μl diluted primary antibody (purified 10 H mouse monoclonal antibody recognising poly [ADP-ribose]; dilution 1:300 in FACS buffer) at 4°C overnight. Cells were then pelleted at 652 *g*, 4°C, for 2 minutes and washed in 240 μl of FACS buffer, as before. The resultant cell pellet was resuspended in 240 μl Alexa 488-conjugated secondary antibody (dilution 1:1000 in FACS buffer; Invitrogen) in the dark followed by shaking at room temperature for 30 minutes, pelleted and washed once more and finally resuspended in 240 μl FACS buffer and left on ice prior to flow cytometry analysis.

Cell samples were analysed by flow cytometry in a FACS Calibur II (Becton Dickinson Immunocytometry Systems) operating with CellQuest version 3.3 software. The total population of viable cells was gated to their forward and side scatter. Control samples were evaluated in each experiment. The fluorescence of untreated cells was recorded to determine the level of background fluorescence for negative control cells, while treated cells with primary and secondary antibody staining were used as positive control cells. A total of 10,000 event files for each sample were acquired individually in "live-gate" mode. Data are expressed as mean fluorescence intensity (MFI) above background.

### FACS-based PARP-activity assay – Protocol B

This method is similar to Protocol A, except for the following modifications: Ethanol, rather than digitonin, is used for permeabilisation; the reaction time (incubation with NAD^+ ^and activator oligonucleotide) was extended to 10 min and this was followed by a second fixation using formaldehyde. For convenience this protocol (which is the recommended version, see Discussion) is described here in a step-by-step fashion:

• Centrifuge cells (2 × 10^5 ^per data point) in 15 ml tube for 5 min at 326 *g *(note that about 1.5 × 10^6 ^PBMC can be recovered from 1 ml of blood)

• Resuspend the pellet in 10 ml PBS

• Count the cells

• Centrifuge the cells for 5 min at 326 *g*

• Resuspend pellet in 100% ethanol (ice-cold)

• Leave at -20°C for at least 20 min

• Centrifuge the cells for 10 min at 815 *g*

• Resuspend the cell pellet in ~ 10 ml buffer A (10 mM Tris-HCl pH 7.8, 1 mM EDTA, 4 mM MgCl_2_, 30 mM 2-mercaptoethanol)

• Centrifuge the cells for 10 min at 815 *g*

• Resuspend pellet in N × 27 μl buffer A (N = number of wells to be filled, each comprising 2 × 10^5 ^cells) and transfer 27 μl per well into a V-shaped 96-well plate

• Incubate the plate with the cells on ice for at least 5 min

• Add 20 μl 3× reaction buffer (with or without NAD^+^) plus 13 μl of 15 mM NaCl incorporating or not activator oligonucleotide (final concentration: 5 μg per reaction) thus reaching a final volume of 60 μl

• Leave at 37°C for 10 min

• Second fixation:

∘ Add 60 μl of 4% formaldehyde in PBS and incubate for at least 20 min at room temperature

∘ add 60 μl of 100 mM glycine in PBS to quench formaldehyde

• Centrifuge for 10 min at 815 *g*

• Wash with 200 μl FACS buffer

• Centrifuge for 5 min at 815 *g*

• Resuspend the pellet in 100 μl primary antibody (10 H) diluted in FACS buffer (final concentration 5 μg/ml)

• Incubate for 1 h at 37°C or overnight at 4°C

• Centrifuge for 5 min at 815 *g*

• Wash twice (as before)

• Centrifuge for 5 min at 815 *g*

• Resuspend pellet in 100 μl of diluted secondary antibody (Alexa 488-conjugated goat anti-mouse, dilution 1:1,000 in FACS buffer) N.B. This and the subsequent steps should be done under subdued light.

• Incubate for 30 min at 37°C

• Wash twice (as before)

• Centrifuge for 5 min at 815 *g*

• Resuspend the pellet in 200 μl FACS buffer

• Leave on ice until FACS analysis (as described above) or store the samples at 4°C

## Competing interests

The author(s) declare that they have no competing interests.

## Authors' contributions

AL, DL, KA, MM, BT, and PH performed the experimental work shown in this paper. AB conceived the study and directed the experimental work. YB participated in the design and coordination of the experiments and helped to draft the manuscript. All authors read and approved the final manuscript.
